# Keratinolytic activities of alkaliphilic *Bacillus* sp. MBRL 575 from a novel habitat, limestone deposit site in Manipur, India

**DOI:** 10.1186/s40064-016-2239-9

**Published:** 2016-05-11

**Authors:** Pintubala Kshetri, Debananda S. Ningthoujam

**Affiliations:** Microbial Biotechnology Research Laboratory, Department of Biochemistry, Manipur University, Canchipur, 795003 India

**Keywords:** Feather-degrading, Keratinase, Alkaliphilic, Hundung, *Bacillus*

## Abstract

Microbial degradation of keratinous wastes is preferred over physicochemical methods as the latter is costlier and not eco-friendly. Novel habitats are promising for discovery of new microbial strains. Towards discovery of novel keratinolytic bacteria, screening of bacterial strains from a novel limestone habitat in Hundung, Manipur, India was done and a promising isolate, **MBRL 575**, was found to degrade native chicken feather efficiently. It could grow over a broad pH range (Langeveld et al. in J Infect Dis 188:1782–1789, 2003; Park and Son in Microbiol Res 164:478–485, 2009; Zaghloul et al. in Biodegradation 22:111–128, 2011; Takami et al. in Biosci Biotechnol Biochem 56:1667–1669, 1992; Riffel et al. in J Biotechnol 128:693–703, 2007; Wang et al. in Bioresour Technol 99:5679–5686, 2008) and in presence of 0–15 % NaCl. Based on phenotypic characterization and 16S rRNA gene sequence analysis, the new keratinolytic limestone isolate was identified as *Bacillus* sp. MBRL 575. It produced 305 ± 12 U/ml keratinase and liberated 120 ± 5.5 mg of soluble peptides and 158 ± 4 mg of amino acids per gram of feather after 48 h of incubation at 30 °C in chicken feather medium. The strain could also degrade feathers of other species besides chicken. The cell-free enzyme was also able to degrade feather. Citrate and soybean meal were found to be the best carbon and nitrogen supplements for enhanced enzyme, soluble peptide and amino acid production. In addition to keratinolytic activity, MBRL 575 also exhibited antagonistic activity against two major rice fungal pathogens, *Rhizoctonia oryzae*-*sativae* (65 %) and *Rhizoctonia solani* (58 %).

## Background

Keratin is the most abundant protein in epithelial cells and forms major components of skin, hair, nail, feather and wool. Keratins are grouped into hard keratins, found in appendages such as feather, hair, hoof and nail and soft keratins found in callus and skin. The former have high disulfide bond content and are tough and inextensible whereas soft keratins such as skin and callus have low content of disulfide bridges and are more pliable (Gupta and Ramnani 2006). 90 % of feather weight is constituted by keratin and feathers are produced in large amounts as waste byproducts of poultry processing plants. Several million tons of feather wastes are produced annually worldwide and such wastes could lead to environmental problems (Xu et al. 2009; Sangali and Brandelli 2000; Jayalakshmi et al. 2012). Feathers are highly recalcitrant to digestion by common proteolytic enzymes such as trypsin, pepsin and papain (Gradisar et al. 2005). The mechanical stability of keratin depends on the tight packaging of proteins in α-helix (α-keratin) or β-sheet (β-keratin) structures and their high degree of cross linkages. Feathers which are hydrolyzed by mechanical or chemical treatment can be converted to animal feed, fertilizer, amino acids, glues and foils. This process not only consumes energy but also degrades some essential amino acids (Cao et al. 2009). Because of these drawbacks, the use of keratinolytic enzymes in the production of amino acids and peptides is becoming attractive for biotechnological applications. Keratinases also find applications in detergent, cosmetic, pharmaceutical, and leather industries. Keratinases capable of degrading prion (PrP^sc^), infectious protein agent causing ‘Mad Cow disease’ in animals, have been reported (Langeveld et al. 2003). So far, keratinases have been reported from species of *Bacillus* (Park and Son 2009; Zaghloul et al. 2011; Takami et al. 1992), *Chryseobacterium* (Riffel et al. 2007; Wang et al. 2008), *Fervidobacterium* (Friedrich and Antranikian 1996; Nam et al. 2002), *Kocuria* (Vidal et al. 2000; Bernal et al. 2006), *Lysobacter* (Allpress et al. 2002) *Microbacterium* (Thys and Brandelli 2006), *Nesterenkonia* (Bakhtiar et al. 2002), *Pseudomonas* (Sharma and Gupta 2010; Tork et al. 2010), *Stenotrophomonas* (Cao et al. 2009; Fang et al. 2013)*, Streptomyces* (Bockel et al. 1995; Cheng et al. 2010; Letourneau et al. 1998; Chitte et al. 1999), *Vibrio* (Xu et al. 2009; Grazziotin et al. 2007), and *Xanthomonas* (Jeong et al. 2010a). However, only a few strains or enzymes have reached commercial exploitation. Keratinases from *Bacillus* spp., particularly *B.licheniformis* and *B.subtilis*, have been extensively studied (Gupta and Ramnani 2006). In this paper, we report the isolation and characterization of an efficient chicken feather degrading Gram positive, endospore forming, alkaliphilic *Bacillus* sp. MBRL 575 which also exhibits antagonistic activity against *Rhizoctonia spp.*

## Materials and methods

### Isolation of the bacterial strains

The soil samples collected from limestone quarry at Hundung, Manipur, India (25.05°N, 94.33°E) were dried and crushed by mortar and pestle. 1 g soil sample was dissolved in 100 ml sterile distilled water and kept incubated in a shaker (25 °C, 150 rpm, 30 min). The soil suspension was serially diluted (10^−3^–10^−7^) and 0.1 ml diluted soil suspension was spread plated on Horikoshi Medium 1 (Horikoshi 2004) and the plates were kept incubated at 30 °C for a week. Morphologically distinct bacterial colonies were selected and subcultured till pure cultures were obtained. The purified cultures were preserved as slants at 4 °C and as glycerol stocks (20 %, v/v) at −20 °C. Bacterial strains were screened for feather degradation in chicken feather medium (CFM) containing feather, 1 % (w/v); KH_2_PO_4_, 0.5 %(w/v); MgSO_4_, 0.05 %(w/v) and Na_2_CO_3_, 0.5 % (w/v). The most promising strain was selected for further studies.

### Identification of the potent feather degrading strain

Phenotypic and biochemical properties of the isolate were investigated according to Cappuccino and Sherman (Cappuchino and Sherman 1999). The strain MBRL 575 was identified on the basis of 16 S rRNA gene sequence analysis. The 16S rRNA gene was amplified using the primers 8F (5-CAGAGTTTGATCCTGGCT-3) and **1522R** (5-AGGAGGTGATCCAGCCGCA-3). The PCR mixture contained 25 μl 2X Mastermix (Promega), 1 μl of each primer (20 mM) and the final volume was made up to 50 μl with deionized water. PCR was carried out with initial denaturation at 94 °C for 5 min followed by 3 cycles of denaturation at 94 °C for 30 s, annealing at 55 °C for 30 s, extension at 72 °C for 90 s and final extension at 72 °C for 10 min. The amplified PCR product was detected by horizontal gel electrophoresis in 1 % agarose gel at 100 V for 90 min using 1X TE buffer. The amplified 16S rRNA gene product was outsourced for sequencing at Xcelris Labs Ltd, India. The sequence obtained was then assembled and submitted to EzTaxon-e server database (Kim et al. 2012). The phylogenetic tree using neighbor-joining algorithm was constructed using MEGA 5 software package (Tamura et al. 2011).

### Inoculum preparation

A loopful of bacterial culture was inoculated in Horikoshi-I broth and kept incubated under shaking conditions (30 °C, 150 rpm, 24 h). The culture broth was centrifuged (10,000 rpm, 30 min). The pellet was collected, washed twice with sterile distilled water and then centrifuged. The pellet was then dissolved in 10 ml sterile distilled water and optical density (OD) was measured at 600 nm and OD was adjusted to 0.5.

### Source of chicken feather

Feathers of 45 day old broiler chickens were collected from local slaughter houses, washed thoroughly with tap water, and dried in the oven. The dried feathers were cut into pieces (2–3 cm) and used for media preparation.

### Feather weight loss

Feather weight loss was measured according to Bertsch and Coello (Bertsch and Coello 2005) with some modifications. The culture broth was filtered through a sieve (1 mm mesh) and washed twice with distilled water. The residue was dried on a pre-weighed filter paper in an oven at 60 °C until constant weight.

### Chicken feather degradation by MBRL 575

Feather degradation was carried out in CFM. The medium was inoculated with 5 % (v/v, 0.5 OD _600_) inoculum. The inoculated flasks were kept incubated on a shaker (150 rpm, 30 °C). Time course of feather degradation was monitored by taking the fermentation broth at various time intervals and monitoring feather weight loss, keratinase, soluble peptide and amino acid production. The strain was also checked for its ability to degrade various bird feathers other than chicken feather by replacing chicken feather with other bird feathers. In order to investigate the effects of heat treatment of feather on keratinase, soluble peptide and amino acid production, chicken feather was autoclaved for different cycles (1, 2 and 3). In another case, feather was surface sterilized with isopropanol instead of autoclaving. Effect of feather concentration was monitored by adding feather at different concentrations. Effects of incubation temperature were also studied by growing MBRL 575 in CFM at different temperatures. For evaluating effects of carbon and nitrogen supplements on feather degradation, various carbon sources viz. glucose, sucrose, citrate, corn starch and lactose were added in CFM at 1 % (w/v) concentration and nitrogen sources viz. peptone, yeast extract, soybean meal, beef extract, KNO_3_ and NH_4_Cl were added at 0.5 % (w/v) concentration.

### Keratinase assay

Keratinase activity was determined using keratin azure as substrate according to Sousa et al. (2007) with some modifications. 20 mg keratin was suspended in 4 ml of 50 mM Glycine-NaOH buffer, pH 10. 1 ml of appropriately diluted enzyme was added and incubated at 55 °C in a water bath for 1 h. The reaction mixture was filtered through glass wool and absorbance was measured at 595 nm. One unit of keratinolytic activity was defined as the amount of enzyme that led to an increase in absorbance of 0.01 at 595 nm under standard assay conditions.

### Determination of soluble peptides and amino acid concentration

Soluble peptide content was determined according to Lowry method using bovine serum albumin (BSA) as standard (Lowry et al. 1951). Amino acid content was measured by ninhydrin method with l-leucine as standard (Lee and Takahashi 1966). In all the experiments, un-inoculated samples were included as controls and used as blanks. Absorbance was measured against the appropriate blank.

### Determination of free thiol group

Free thiol group concentration was measured using Ellman reagent [DNTB (5,5′-dithio (2-nitrobenzoic acid)] with reduced glutathione as standard. 39.6 mg DTNB was dissolved in 10 ml phosphate buffer (100 mM, pH 7.2). To 3 ml of appropriately diluted sample, 20 μl DTNB solution was added. A blank sample (un-inoculated medium) was also included in the reaction. Then, the absorbance was measured at 412 nm after 2 min of stable colour development against the blank (Ellman 1959).

### Preparation of concentrated enzyme extract

To 1.0 L enzyme extract, 390 g of anhydrous (NH_4_)_2_SO_4_ was added (60 % saturation) and the mixture was centrifuged (10,000 rpm, 30 min). The precipitate obtained was dissolved in a minimal volume of Glycine-NaOH buffer (50 mM, pH 10) and dialyzed overnight against the same buffer at 4 °C.

### Release of soluble peptides from native feather by cell free enzyme extract

1 g of non autoclaved and autoclaved chicken feathers (1 cycle autoclaved) were treated with 100 ml of concentrated enzyme extract (600 U/ml) and incubated at 40 °C. The reaction mixture was taken out at various time intervals and the enzyme was then inactivated by heating in boiling water bath. Then, the reaction mixture was filtered through a Whatman no. 1 filter paper and release of soluble peptides by keratinase enzyme was monitored.

### Evaluation of biocontrol activity

The strain MBRL 575 was subjected to biocontrol assay against major rice fungal pathogens (*Rhizoctonia solani* and *Rhizoctonia oryzae*-*sativa*) on nutrient agar plates by dual culture technique. Colony growth inhibition was calculated using the formula:

$${\text{Percentage}}\,{\text{of}}\,{\text{colony}}\,{\text{growth}}\,{\text{inhibition}} = [({\text{C}} - {\text{T}})/{\text{C}}] \times 100$$

where, C represents the radial growth of the test pathogen in the control plates (mm), and T is the radial growth of the test pathogen in the test plates (mm) (Hamdali et al. 2008).

### Scanning electron microscopy

MBRL 575 grown in CFM and fermentation broth was filtered with Whatman No.3 filter paper after 0 h, 12 h and 24 h of incubation. The filtered feather was fixed with 2.5 % (v/v) glutaraldehyde and 2 % (v/v) formalaldehyde for 24 h. The specimens were dehydrated several times with 70–80 % acetone and dried at 50 °C for 10 min. The specimens were observed using FEI Quanta 250 scanning electron microscope (Rahayu et al. 2012).

### Statistical analysis of data

All the above experiments were repeated in triplicate and the final values have been presented as mean ± S.D.

## Results and discussion

### Isolation and identification of alkaliphilic bacterial strains

16 morphologically distinct bacterial strains were recovered from soil samples collected from limestone deposit sites. The isolates were investigated for possible degradation of chicken feather in CFM medium. 3 strains were found to degrade chicken feather. One isolate, MBRL 575, could degrade feather completely in 48 h. However, the other two isolates (UAH-5 and UAH-7) took 4–5 days for complete degradation of feather. Hence, MBRL 575 was selected for further studies.

**Fig. 1:**
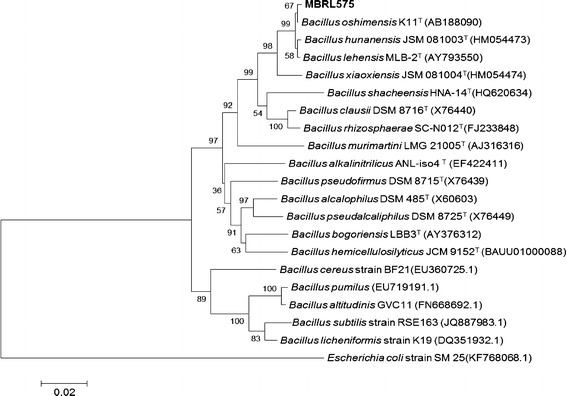
Neighbour-joining tree based on 16S rRNA gene sequences, showing the relationships between strain MBRL 575 and other type strains of *Bacillus* species. *Escherichia coli* strain SM 25(KF768068.1) was used as the outgroup. Numbers at nodes are levels of bootstrap support (%) for branch points (1000 resamplings). *Bar* 0.02 substitutions per nucleotide position

The strain MBRL 575 is a gram positive, endospore forming, rod shaped, motile bacterium. It showed positive results for catalase, oxidase, nitrate reduction, methyl red, gelatin liquefaction, and starch hydrolysis tests and negative for, Voges-Proskauer, Tween 20/80 hydrolysis and urease production tests. It could produce acid from glucose, fructose, sucrose, melibiose, mannitol, trehalose, mannose, lactose and inulin but not from glycerol and galactose. The organism forms opaque yellow colonies on Horikoshi Medium 1; colonies were regular in shape, moist and very sticky. It could grow at temperature range of 20–45 °C and tolerate up to 15 % NaCl. It grew at pH 7–12 with an optimum pH of 9–10. Based on morphological characterization and 16S rDNA sequence analysis, MBRL 575 was identified as a *Bacillus* sp. and designated as *Bacillus* sp. MBRL 575(NCBI GenBank Accession No. **KC865830**). It showed closest 16S rRNA gene sequence similarities with *Bacillus oshimensis* (99.63 %), *Bacillus lehensis* (99.56 %) and *Bacillus hunanensis* (99.34 %). Nimaichand et al. (Nimaichand et al. 2015) have earlier reported isolation of actinobacteria from limestone deposit site at Hundung but this is the first report on isolation of alkaliphilic *Bacillus* species from this region. Analysis of phylogenetic tree indicated that MBRL 575 is not closely related with other feather degrading *Bacillus* spp. e.g. *B. licheniformis* (Xu et al. 2009), *B.subtilis* (Gupta and Singh 2014) *B.cereus * (Lakshmi et al. 2013*), B.pumilus* (Fakhfakh-Zouari et al. 2010)*, and B.altitudinis* (Kumar et al. 2011*)* which have been reported earlier (Fig. 1).

### Time course of feather degradation

**Fig. 2:**
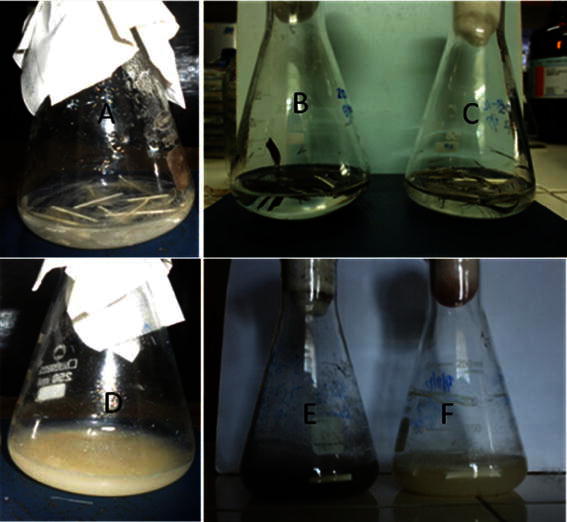
Degradation of different bird feathers by *Bacillus* sp. MBRL 575 at 48 h, 30 °C. **a** Chicken feather (control), **b** duck feather (control), **c** Turkey feather (control), **d** chicken feather (48 h incubation), **e** duck feather (48 h incubation) **f** Turkey feather (48 h incubation)

**Fig. 3:**
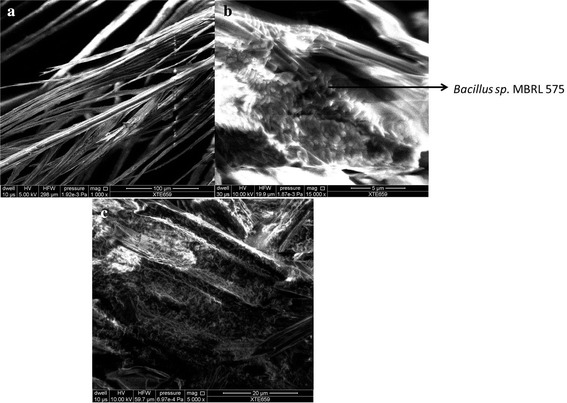
Scanning electron micrograph of feather. **a** Control (untreated feather). **b** Feather degradation by *Bacillus* sp. MBRL 575 after 12 h of incubation. **c** Feather degradation by *Bacillus* sp. MBRL 575 after 24 h of incubation

**Fig. 4:**
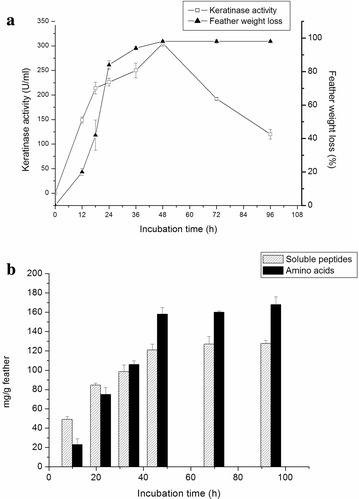
Time course of feather degradation by MBRL 575. **a** Feather weight loss and keratinase production as a function time. MBRL 575 was grown in CFM and measured the keratinase activity and feather weight loss at 12 h time interval. Each point represents the mean of three independent experiments. **b** Release of soluble peptides and amino acids by MBRL 575 at 12 h time intervals. Cell-free supernatant was used for the measurement of soluble peptides by the Folin’s method and amino acids by ninhydrin method. Each point represents the mean of three independent experiments

Degradation of chicken and other bird feathers by strain MBRL 575 are shown in Fig. 2. Degradation of feather barbules was apparent from 12 h of incubation onwards. However, complete degradation (98 % weight loss) was observed after 48 h at 30 °C. According to a previous report by El-Refai et al. different *Bacillus* spp. showed different extents of feather degradation as indicated by feather weight loss viz. *Bacillus pumilus* FH9 (96 %), *Bacillus licheniformis* SA1 (70.8 %) and *Bacillus subtilis* (42.0 %) when grown on basal medium supplemented with 1 % chicken feathers after 48 h of incubation 37 °C (El-Refai et al. 2005). Nagal and Jain reported that *Bacillus cereus* KB043 showed 78.16 ± 0.4 % weight loss after 6 days of incubation at 37 °C (Nagal and Jain 2010). Park and Son reported complete degradation of chicken feather by *Bacillus megaterium* after 7 days (Park and Son 2009) and Williams et al. (1990) showed that *Bacillus licheniformis* PWD-1 degraded chicken feather completely after 10 days. In comparison to the feather degrading bacteria mentioned earlier, MBRL 575 is a relatively more efficient feather degrading strain. Scanning electron microscopic (SEM) observations revealed the colonization of bacteria on feather as well as disintegration of feather barbules after 12 h of incubation (Fig. 3b). After 24 h of incubation, the rachi and barbules were degraded and bacteria were found embedded in biomass of the degraded feathers (Fig. 3c). MBRL 575 produced 305 ± 12 U/ml keratinase and could liberate 120 ± 5.5 mg of soluble peptides and 158 ± 4 mg of amino acids per gram of feather after 48 h of incubation (Figs. 4a, b). Laba and Rodziewicz reported that 2.1 mg/ml (210 mg/g of feather) of soluble protein was liberated by *Bacillus polymyxa* and 2.35 mg/ml (235 mg/g of feather) by *Bacillus cereus* after 10 days of incubation (Laba and Rodziewicz 2010). Production of soluble protein and amino acids during feather degradation by various bacterial species have been reported earlier viz. *Bacillus subtilis* (Jeong et al. 2010b; Zaghloul et al. 2011), *Bacillus pumilus* (Son et al. 2008), *Stenotrophomonas maltophilia* (Jeong et al. 2010c), and *Chryseobacterium* sp. kr 6 (Riffel et al. 2003). Feather degradation is always accompanied by release of soluble peptides and amino acids in the culture medium. The major fraction of soluble protein present in the culture broth comes from feather keratin while the rest is accounted for by secretion of enzyme/protein by the microbe. Some part of the solubilized feather keratin is converted to microbial biomass and some are hydrolyzed to amino acids by the action of proteases (Daroit et al. 2009). Hence, extent of release of soluble protein and amino acids varies from organism to organism.

### Degradation of different bird feathers

**Fig. 5:**
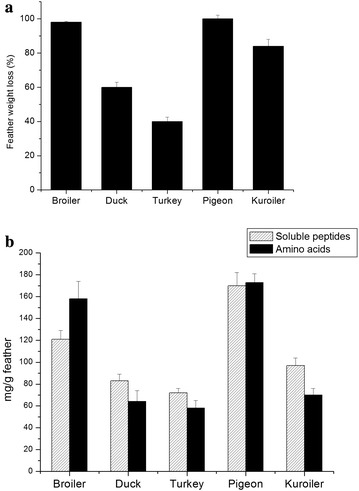
Degradation of various bird feathers by MBRL 575. **a** Comparison of degradation of different bird feathers by *Bacillus* sp. MBRL 575 (feather weight loss). MBRL 575 was grown in medium containing 1 % different feathers and kept incubated at 30 °C, 150 rpm for 48 h. Each point represents the mean of three independent experiments. **b** Release of soluble peptides and amino acid production per gram of different bird feathers by *Bacillus* sp. MBRL 575 after 48 h of incubation. Each point represents the mean of three independent experiments

MBRL 575 could degrade several other bird feathers other than chicken feather. Feather weight loss, keratinase, soluble peptide and amino acid production occurred in the decreasing order of pigeon > broiler > kuroiler > duck > turkey (Fig. 5a, b). Similar results were also reported by Saber et al. who observed chicken feather to be most efficiently degraded among various bird feathers (duck, goose, turkey) by two fungi *Alternaria tenuissima* and *Aspergillus nidulans* (Saber et al. 2009). MBRL 575 could degrade pigeon and broiler feather completely within 48 h of incubation. 84 % feather weight loss was observed in kuroiler feather, 60 % for duck feather and 40 % for turkey feather. This showed that white feathers are more easily degraded than the coloured feathers. Resistance to bacterial degradation by coloured feathers may be attributed to presence of melanin pigment as observed by Gunderson et al. (2008). Efficient degradation of chicken feather by MBRL 575 is an important feature from bio-industrial point of view as chicken is most commonly consumed over other birds all over the world. Moreover, the ability of the organism to degrade different bird feathers may be a positive feature for bio-waste management.

### Effect of various factors on feather degradation

As physiological and nutritional factors greatly affect feather degradation and keratinase production, the effects of various factors such as pretreatment of feather, feather concentration, incubation temperature and supplementary carbon and nitrogen sources on feather degradation by MBRL 575 were also studied and the results are summarized in Tables 1 and 2. Autoclaving of feather accelerated degradation of feather and production of keratinase, soluble peptides and amino acids. Only 46 % feather weight loss was achieved by MBRL 575 in non-autoclaved feather as compared to 98 % loss in autoclaved feather. Enhancement of feather degradation by autoclaving may be attributed to splitting of hydrogen bonds in keratin fibers by heating during autoclaving which make the keratin fibers more accessible to keratinase action. Similar results were also demonstrated by Szabo et al. who reported that heat sterilized feathers were digested more efficiently than γ- ray or ethylene oxide sterilized feathers (Szabo et al. 2000). MBRL 575, interestingly, could degrade very high concentrations of feather after 48 h of incubation at 30 °C. Even at 10 % (w/v) feather concentration, 40 % of feather weight loss was achieved. Maximum keratinase production (305 ± 12 U/ml) was observed at 1 % feather concentration and further increase in feather concentration led to decreased keratinase production. Kainoor and Naik also observed that maximum keratinase production by *Bacillus* sp. JB 99 occurred at 1 % feather concentration and further increase in feather concentration led to decreased enzyme production (Kainoor and Naik 2010). Cheng et al. 1995 also reported that higher concentration of feather powder decreased keratinase production by *B. licheniformis* PWD 1. Soluble peptide and amino acid production by MBRL 575 increased as the feather concentration increased. However, release of soluble protein or amino acids per gram of feather was higher at lower feather concentrations and decreased as the feather concentration increased. Presence of high concentrations of feather in fermentation medium leads to drastic decrease in aeration and increase in viscosity which, in turn, retards microbial growth and keratinase production (Ramnani and Gupta 2004). Incubation temperature profoundly influenced keratinase production. Incubation temperature of 30 °C resulted in maximal enzyme, soluble peptide and amino acid production. No feather degradation was observed at 40 °C. Many keratinolytic bacteria such as *B. megaterium* F7-1, *B. subtilis* and *Chryseoobacterium* strain kr 6 showed optimum keratinase production at moderate temperatures (Park and Son 2009; Jeong et al. 2010b; Saber et al. 2009). Keratinolytic activity of mesophilic organisms may be an interesting property for biotechnological application as these microorganisms will be less energy consuming than the thermophilic ones for feather waste digestion (Jeong et al. 2010b).

**Table 1:** Effect of different factors on feather degradation by MBRL 575

Effect/treatment	Feather weight loss (%)	Keratinase activity (U/ml)	Soluble peptides (mg/g Feather)	Amino acids (mg/g feather)
Temperature (°C)				
25	41 ± 3.6	80 ± 2	100 ± 2.5	109 ± 3.6
28	79 ± 3.6	268 ± 4.04	111 ± 3.05	123 ± 3.6
30	98 ± 0.4	305 ± 12	120 ± 5.5	158 ± 4
35	48 ± 2	95 ± 3.05	102 ± 6.1	108 ± 2
Feather concentration (w/v %)				
0.5	100 ± 0.5	145 ± 7	161 ± 3	180 ± 6
1.0	98 ± 0.4	305 ± 12	120 ± 5.5	158 ± 4
2.0	85 ± 1	237 ± 11	110 ± 2.6	135 ± 2.5
3.0	76 ± 2	139 ± 7.7	98 ± 2	116 ± 3.05
5.0	61 ± 2.5	104 ± 6.02	80 ± 2.5	94.3 ± 4.04
7.0	53 ± 2.5	77 ± 7.5	81 ± 5.56	94 ± 3
10	45 ± 3	32 ± 4	81.3 ± 6.4	90 ± 2
Pre-treatment of feather				
Non-autoclaved	46 ± 4	107 ± 8	58 ± 8.2	98 ± 2
One cycle autoclaved	98 ± 0.5	305 ± 12	120 ± 5.5	158 ± 4
Two cycle autoclaved	98 ± 2	310 ± 10	122 ± 4	162 ± 6
Three cycle autoclaved	99 ± 3	316 ± 7	126 ± 6.2	160 ± 8.2

**Table 2:** Effect of carbon and nitrogen supplements on keratinase, soluble peptides and amino acid production

	Keratinase (U/ml)	Soluble peptides (mg/g feather)	Amino acids (mg/g feather)
Carbon source (1 %, w/v)			
Control^a^	305 ± 12	120 ± 5.5	158 ± 4
Glucose	452 ± 6	136 ± 7	168 ± 14
Na Citrate	482 ± 16	150 ± 8.5	227 ± 18
Corn starch	418 ± 20	130 ± 4	176 ± 8
Lactose	280 ± 10	88 ± 6.2	102 ± 6.4
Sucrose	394 ± 14	129 ± 12	154 ± 2.4
Nitrogen source (0.5 %, w/v)			
Peptone	350 ± 8	130 ± 6	176 ± 6
Yeast extract	338 ± 10	107 ± 16	130 ± 14
Soybean meal	536 ± 24	164 ± 10	263 ± 8
Beef extract	362 ± 6	125 ± 10.2	173 ± 12
KNO_3_	209 ± 8	115 ± 8	54 ± 6
NH_4_Cl	17 ± 3	52 ± 4	87 ± 4

^a^Control represents CFM medium without any supplementary carbon or nitrogen source

### Effect of supplementary carbon (C) and nitrogen (N) sources

Among the C sources tested, sodium citrate was found to be the best C supplement for keratinase production (482 ± 16 U/ml) followed by glucose (452 ± 6 U/ml), corn starch (418 ± 20 U/ml) and sucrose (394 ± 14 U/ml). Johnvesly and Naik (2001) also reported trisodium citrate and citric acid as good C sources for enhanced alkaline protease production. The use of these organic acids is interesting in view of their economy as well as their ability to control pH variation (Kumar and Takagi 1999). In contrast to our results, Kainoor and Naik reported that citric acid and other carbon sources led to decreased enzyme production by *Bacillus* sp. JB 99 (Kainoor and Naik 2010). Enhancement of keratinase production by glucose was also reported for *Bacillus licheniformis ER*-*15* (Tiwary and Gupta 2010), *Bacillus subtilis* (Jeong et al. 2010b) and *Bacillus megaterium* F7-1 (Park and Son 2009). Addition of complex N sources such as soybean meal (536 ± 24 U/ml), beef extract (362 ± 6 U/ml), peptone (350 ± 8 U/ml) and yeast extract (338 ± 10 U/ml) enhanced keratinase production by MBRL 575. Cheng et al. reported that supplementation of soybean meal enhanced keratinase production by *B. licheniformis* PWD 1(Cheng et al. 1995). In some organisms, soy flour was reported as good N supplement for keratinase production (Gradisar et al. 2005; Tiwary and Gupta 2010). However, inorganic nitrogen sources such as KNO_3_ and NH_4_Cl led to decreased enzyme production by MBRL 575. Similarly, Sahoo et al. (2012) reported inhibition of keratinase production by NH_4_Cl and KNO_3_. The inhibitory effect of inorganic salts on keratinase production may be attributed to the release of ammonia from these inorganic nitrogen sources (Uyar et al. 2011). Moreover, supplementation of C and organic N sources also enhanced production of soluble protein and amino acids by MBRL 575.

### Determination of free thiol group

**Fig. 6:**
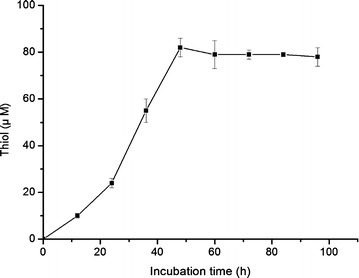
Free thiol group released by MBRL 575. MBRL 575 was grown in CFM at 30 °C, 150 rpm. Fermentation broth was collected at 12 h time interval and thiol production was measured using Ellman’s reagent. Each point represents the mean of three independent experiments

Thiol formation increased during the exponential phase of microbial growth reaching maximum (82 μM) at 3 days of incubation (Fig. 6). This coincides with the maximum enzyme production period. Microbial degradation of keratin is a complex process and one of the possible mechanisms for keratin degradation is the reduction of disulphide bonds. The mechanism of keratin degradation by strain MBRL 575 might also involve sulphitolysis as it produced free thiol group during keratin degradation. Similar reduction of disulfide bonds during feather degradation was also observed for *Streptomyces pactum,Bacillus subtilis* and *Cryseobacterium* sp. *strain 6* (Bockel et al. 1995; Jeong et al. 2010b; Riffel et al. 2003).

### Feather degradation by cell free enzyme extract

**Fig. 7:**
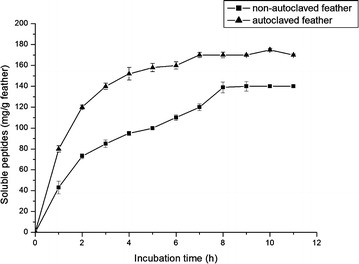
Release of soluble peptides by cell free concentrated enzyme extract. 1 g feather was treated with 100 ml (glycine-NaOH buffer pH 10) concentrated enzyme (600 U/ml) and kept incubated at 40 °C. Aliquots are taken out at 1 h time interval and measured release of soluble peptide using Folin’s reagent. Each point represents the mean of three independent experiments

During fermentation soluble peptides and essential amino acids released from feather are utilized by the microorganism which decreases the nutritional value of feather meal (Tiwary and Gupta 2012). To circumvent this, research is now focused on use of keratinases rather than live microbial cells for feather meal preparation. Hence, the cell free enzyme extract of MBRL 575 was evaluated for feather degradation. The enzyme extract could release soluble peptides from native feathers (Fig. 7). Similarly, Vasela and Friedrich (2009) reported that crude keratinase from *Paecilomyces marquandii* released soluble peptides from keratin substrates. In their case, release of soluble peptides increased till 4 h of incubation and remained constant. However, in our study release of soluble peptides increased till 7 h of incubation. Moreover, MBRL 575 keratinase could also degrade non-autoclaved chicken feathers. Soluble peptide release was higher in autoclaved (170 mg/g feather) feathers as compared to non-autoclaved feathers (140 mg/g feather). In contrast, Park and Son reported that the cell free enzyme extract of *Bacillus megaterium* F7-1 could only degrade autoclaved feather and it could not degrade non autoclaved feather (Park and Son 2009).

### Evaluation of antagonistic activity against *Rhizoctonia* spp. by strain MBRL 575

**Fig. 8:**
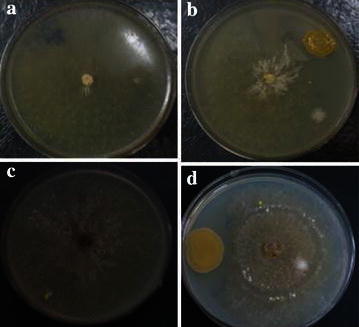
Biocontrol activity of MBRL 575. **a** *Rhizoctonia oryzae*-*sativae* (control) **b** antagonistic activity against *Rhizoctonia oryzae*- *sativae* **c** *Rhizoctonia solani* (control) **d** antagonistic activity against *Rhizzoctonia solani*

Besides efficient keratinolytic activity, the strain MBRL 575 showed antagonistic activity against major rice fungal pathogens, *Rhizoctonia oryzae*-*sativae* and *Rhizoctonia solani*, causing aggregate sheath spot disease and sheath blight respectively (Fig. 8). It showed more than 50 % mycelial growth inhibition of both the indicator strains (Table 3). Some keratinolytic bacterial strains viz. *Xanthomonas* sp. P5, *Bacillus subtilis* and *Stenotrophomonas maltophilia* having antagonistic activity against phytopathogens have been previously reported (Jeong et al. 2010a, b; c).

**Table 3:** Antagonistic activity of MBRL 575 against *Rhizoctonia spp*

Fungal Pathogen	% of fungal mycelial growth inhibition
*R. oryzae*- *sativae*	65
*R. solani*	58

## Conclusions

A new feather degrading alkaliphilic bacterial strain, *Bacillus* sp. MBRL575, was isolated from a limestone habitat in Hundung, Manipur, India. The strain was an efficient feather degrader, achieving nearly complete degradation of broiler feather after 48 h of incubation. The organism produced significant amounts of enzyme, soluble peptides and amino acids in minimal medium containing chicken feather as the sole carbon and nitrogen source. There are meager reports of alkaliphilic *Bacillus* spp. that degrade feather. MBRL 575 is the first alkaliphilic *Bacillus* strain from limestone habitat with keratinolytic activity. None of the alkaliphilic *Bacillus* strains viz. *B. oshimensis, B. lehensis and B. hunanensis*, closely related to MBRL 575 has been reported to have feather degrading activity. Moreover, this strain did not show close phylogenetic affinities with other previously reported feather degrading *Bacillus* spp. The present study will add a new feather degrading alkaliphilic *Bacillus* sp. to the repertoire of keratinolytic bacteria. MBRL 575 and its keratinase are promising candidate agents for biotechnological application in feather waste bioremediation and valorization. In addition, the organism also exhibited antagonistic activities against two major rice pathogens: *R. solani* and *R. oryzae*-*sativae*. Further investigations will be needed to explore the potential of MBRL 575 as a biocontrol and/or biofertilizer agent.
